# Supplementary material for: Biogeographic barriers drive co-diversification within associated eukaryotes of the Sarracenia alata pitcher plant system
Source: PeerJ. 2016 Jan 14;4:e1576. doi: 10.7717/peerj.1576 (PMC4715430; doi:10.7717/peerj.1576)
Supplement: Table S1 — Values represent the number of sequences from the corresponding locale. [file peerj-04-1576-s002.docx]

Table S1. Sampling distribution for each OTU. Values represent the number of sequences from the corresponding locale.

|  | **West** | | **East** | | |
| --- | --- | --- | --- | --- | --- |
| **Code** | **C** | **K** | **A** | **L** | **T** |
| Fungi1 | 0 | 21 | 0 | 26 | 5 |
| Fungi2 | 0 | 7 | 3 | 40 | 1 |
| Fungi3 | 4 | 4 | 2 | 9 | 3 |
| Fungi4 | 270 | 170 | 302 | 1458 | 307 |
| Fungi5 | 7 | 15 | 12 | 50 | 13 |
| Fungi6 | 19 | 12 | 20 | 92 | 25 |
| Fungi7 | 14 | 5 | 12 | 48 | 5 |
| Fungi8 | 7 | 4 | 7 | 35 | 4 |
| Fungi9 | 5 | 2 | 4 | 16 | 3 |
| Fungi10 | 5 | 3 | 5 | 33 | 8 |
| Fungi11 | 18 | 0 | 66 | 74 | 31 |
| Fungi12 | 0 | 9 | 1 | 4 | 0 |
| Fungi13 | 3 | 1 | 2 | 29 | 5 |
| Fungi14 | 73 | 36 | 75 | 463 | 119 |
| Fungi15 | 0 | 5 | 7 | 3 | 0 |
| Amoebozoa1 | 29 | 3 | 32 | 71 | 92 |
| Alveolata1 | 12 | 0 | 0 | 6 | 0 |
| Nematoda1 | 0 | 68 | 8 | 1 | 2 |
| Nematoda2 | 0 | 124 | 29 | 1 | 0 |
| Nematoda3 | 0 | 18 | 3 | 0 | 0 |
| Insect1 | 25 | 13 | 0 | 23 | 0 |
| Insect2 | 9 | 6 | 0 | 18 | 4 |
| Insect3 | 2 | 32 | 0 | 6 | 1 |
| Mite1 | 144 | 71 | 34 | 509 | 70 |
| Mite2 | 2 | 2 | 4 | 21 | 1 |
| Mite3 | 318 | 132 | 173 | 378 | 70 |
| Mite4 | 16 | 5 | 12 | 16 | 7 |
| Mite5 | 17 | 7 | 1 | 5 | 4 |
| Mite6 | 6 | 9 | 11 | 18 | 6 |
| Mite7 | 19 | 7 | 4 | 10 | 5 |
| Unknown | 3 | 1 | 0 | 60 | 2 |
