# Supplementary material for: Biogeographic barriers drive co-diversification within associated eukaryotes of the Sarracenia alata pitcher plant system
Source: PeerJ. 2016 Jan 14;4:e1576. doi: 10.7717/peerj.1576 (PMC4715430; doi:10.7717/peerj.1576)
Supplement: Table S2 — AMOVA analyses show the hierarchical partitioning scheme of locales within regions (ΦSC), locales within total distribution (ΦST), and between regions (ΦCT). Mantel r values are reported for the IBD analyses. Asterisks denote significant values (∗ <0.05–0.01; ∗∗ <0.01–0.001; ∗∗∗ <0.001). [file peerj-04-1576-s003.docx]

Table S2 – Results from AMOVA, GSI and IBD analyses. AMOVA analyses show the hierarchical partitioning scheme of locales within regions (**Φ_SC_**), locales within total distribution (**Φ_ST_**), and between regions (**Φ_CT_**). Mantel *r* values are reported for the IBD analyses. Asterisks denote significant values (* < 0.05 – 0.01; ** < 0.01 – 0.001; *** < 0.001).

| **Taxa** | **Φ_SC_** | **Φ_ST_** | **Φ_CT_** | **GSI_E_** | **GSI_W_** | **IBD** |
| --- | --- | --- | --- | --- | --- | --- |
| Fungi1 | -0.0790 | 0.0171 | 0.0891 | 0.1905 | 0.1026 | 0.8911 |
| Fungi2 | -0.1873 | 0.1595** | 0.2920 | 0.2560* | 0.2647** | 0.6555 |
| Fungi3 | -0.0266 | 0.2030** | 0.2237 | 0.1711 | 0.3750* | 0.6687 |
| Fungi4 | 0.0035*** | 0.0025** | -0.0010 | 0.0200 | 0.0400 | -0.2124 |
| Fungi5 | 0.0369 | 0.0472* | 0.0108 | 0.1848** | 0.0326 | 0.0021 |
| Fungi6 | -0.0021 | -0.0170 | -0.0149 | 0.1077 | 0.1919*** | -0.5135 |
| Fungi7 | 0.0350 | 0.0115 | -0.0243 | 0 | 0.0468 | -0.0657 |
| Fungi8 | -0.0340 | 0.0747* | 0.1051 | 0.0744 | 0.0473 | 0.6378 |
| Fungi9 | 0.1850 | 0.0844 | -0.1235 | 0 | 0.0417 | -0.2010 |
| Fungi10 | 0.0200 | -0.0172 | -0.0380 | 0 | 0.0546 | -0.0680 |
| Fungi11 | -0.0041 | -0.0043 | -0.0001 | 0 | 0.0269 | 0.0450 |
| Fungi12 | 0.5276 | 0.5914*** | 0.1351 | 1*** | 0.7111** | 0.8576 |
| Fungi13 | 0.0277 | 0.0382 | 0.0109 | 0 | 0.0972 | 0.6806** |
| Fungi14 | 0.0063* | 0.0152*** | 0.0089 | 0.0158 | 0.0421 | 0.5492* |
| Fungi15 | -0.0538 | -0.0691 | -0.0145 | 0 | 0.0667 | -0.5888 |
| Amoebozoa1 | 0.0006 | -0.0043 | -0.0048 | 0 | 0.0163 | -0.4741 |
| Alveolata1 | NA | NA | NA | 0.1736 | 0 | NA |
| Nematoda1 | -0.3959 | -0.0960 | 0.2148 | 0.0474 | 0 | -0.0356 |
| Nematoda2 | 0.4389 | 0.0147 | -0.7560 | 0.1037 | 0.0270 | -0.6878 |
| Nematoda3 | NA | NA | NA | 0.1111 | 0 | NA |
| Insect1 | 0.6965*** | 0.4416*** | -0.8399 | 0.1930** | 0.0555 | -0.3276 |
| Insect2 | 0.0548 | 0.0537 | -0.0011 | 0.1750 | 0.0579 | 0.4669 |
| Insect3 | 0.7490*** | 0.4277*** | -1.2799 | 0.1597 | 0.1209 | 0.1049 |
| Mite1 | 0.0099** | 0.0198*** | 0.0100 | 0.1220*** | 0.0773** | 0.1900 |
| Mite2 | -0.1093 | -0.0400 | 0.0625 | 0.2232 | 0.0938 | 0.2335 |
| Mite3 | 0.0098*** | 0.0232*** | 0.0135 | 0.2108*** | 0.1017*** | 0.6383 |
| Mite4 | 0.0181 | 0.0313 | 0.0134 | 0.2361* | 0.1270 | 0.1497 |
| Mite5 | -0.1100 | -0.1131 | -0.0028 | 0.1406 | 0 | 0.3589 |
| Mite6 | 0.1470** | 0.1250** | -0.0257 | 0.1478 | 0.1600 | -0.1140 |
| Mite7 | 0.0447 | 0.0194 | -0.0265 | 0.0507 | 0.0963 | 0.3238 |
| Unknown | -0.1937 | -0.1484 | 0.0379 | 0.0681 | 0 | 0.2020 |
| Host plant | 0.7342*** | 0.9190*** | 0.6952** | 1*** | 1*** | 0.6427** |
